# Contrast-enhanced ultrasonography for the management of portal hypertension in cirrhosis

**DOI:** 10.3389/fmed.2022.1057045

**Published:** 2022-12-14

**Authors:** Hitoshi Maruyama, Maki Tobari, Hiroaki Nagamatsu, Suichiro Shiina, Tadashi Yamaguchi

**Affiliations:** ^1^Department of Gastroenterology, Juntendo University, Tokyo, Japan; ^2^Center for Frontier Medical Engineering, Chiba University, Chiba, Japan

**Keywords:** ultrasound, contrast agent, liver, portal hypertension, cirrhosis

## Abstract

Portal hypertension is a major pathophysiological condition in patients with cirrhosis. This accounts for the occurrence and severity of the various manifestations. The degree is determined by the portal pressure or hepatic venous pressure gradients, both of which are obtained by invasive interventional radiological procedures. Ultrasound (US) is a simple and minimally invasive imaging modality for the diagnosis of liver diseases. Owing to the availability of microbubble-based contrast agents and the development of imaging modes corresponding to contrast effects, contrast-enhanced US (CEUS) has become popular worldwide for the detailed evaluation of hepatic hemodynamics, diffuse liver disease, and focal hepatic lesions. Recent advancements in digital technology have enabled contrast-based demonstrations with improved resolution, leading to a wider range of applications. This review article describes the current role, benefits, and limitations of CEUS in the management of portal hypertension.

## Introduction

Portal hypertension (PH) is the key pathophysiology of cirrhosis (1, 2). The severity of PH is determined by portal venous pressure, which is closely associated with clinical manifestations, such as gastroesophageal varices, portal hypertensive gastropathy, hepatic encephalopathy, and ascites (3). As portal pressure is also a significant prognostic factor, it is recognized as a core factor in the medical care of patients with cirrhosis.

The hepatic venous pressure gradient (HVPG), obtained by hepatic venous catheterization, is a marker alternative to directly measuring portal venous pressure. Although hepatic venous catheterization is less invasive than percutaneous transhepatic portography, a recent trend encourages the application of non-invasive tools that may replace invasive procedures to reduce the burden on patients (4, 5).

Ultrasound (US) is a minimally invasive procedure with the advantage of real-time observation of anatomical structures and hemodynamics under physiological conditions (6). With the availability of microbubble-based contrast agents, contrast-enhanced US (CEUS) has become a popular imaging technique for evaluating liver diseases (7–9). This article focuses on the current concept, effects, and limitations of CEUS in the management of PH in cirrhosis.

## Contrast agents

### Materials

Contrast agents used for US examination are microbubble-based materials. The characteristic properties depend on the type of gas and the shell. It can travel in the bloodstream after intravenous injection because the diameter of the microbubbles is smaller than that of red blood cells.

Levovist is a first-generation contrast agent characterized by galactose-based air-filled microbubbles with palmitic acid (Schering AG, Berlin, Germany) (10). There are three types of newer generations available for abdominal organs (11), namely, SonoVue (sulfur hexafluoride; Bracco, Milan, Italy), Definity (Perflutren Lipid Microsphere; Lantheus Medical Imaging, Billerica, MA, United States), and Sonazoid (perfluorobutane; GE Healthcare United Kingdom Ltd., Pollards Wood, United Kingdom). SonoVue and Definity are blood pool contrast agents that circulate and remain in blood vessels. However, Sonazoid accumulates in reticuloendothelial systems such as Kupffer cells. These second-generation microbubble agents consist of less diffusible gas cores with very flexible and soft envelopes to improve stability and persistence (10).

### Assessment of time-related changes of contrast enhancement

**Figure 1:**
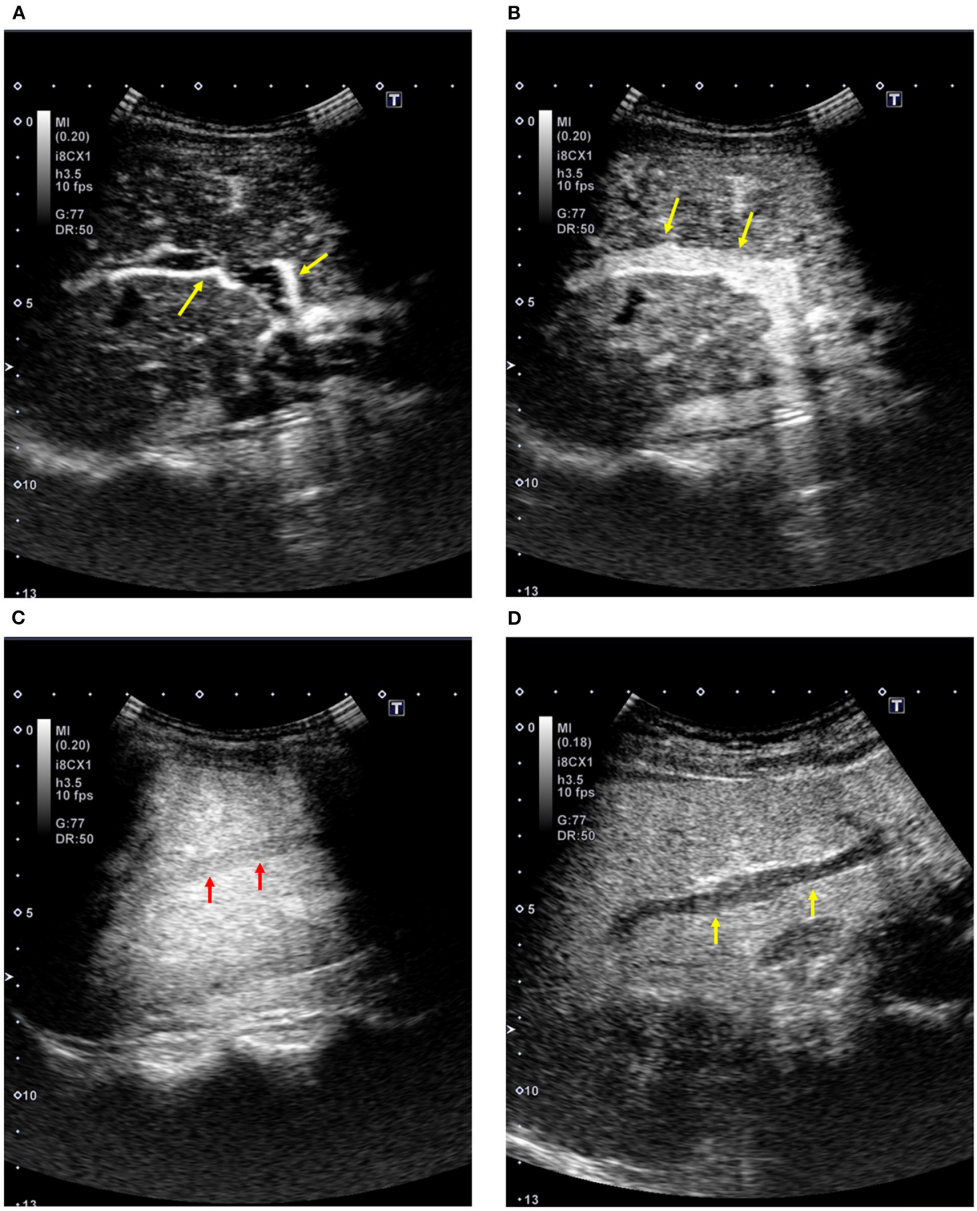
Contrast-enhanced ultrasound of the liver (Sonazoid; healthy subject, 51-year-old women). **(A)** Arterial phase: The sonogram shows an enhancement in the right hepatic artery (arrows). **(B)** Portal venous phase: The sonogram shows an enhancement in the right portal vein (arrows). **(C)** Contrast enhancement in the hepatic vein: The sonogram shows an enhancement in the right hepatic vein (arrows). **(D)** Post-vascular phase (12 min after the injection): The sonogram shows homogeneous enhancement in the liver parenchyma with the absence of enhancement in the intrahepatic vessels (arrows).

The contrast effect changes over time with the circulation of the contrast agent after intravenous injection. Intrahepatic contrast enhancement shows time-related changes due to dual vascular supply by the hepatic arteries and portal veins. When the contrast effect in the liver is assessed by a low-mechanical index (MI) setting that enables the minimal breakdown of microbubbles, the phases show the following in a physiological manner (Figures 1A–D): arterial phase from 10–20 s to 30–50 s, portal venous phase from 30–50 s to 120 s, and late phase after 120 s to the time of microbubble disappearance (Table 1) (11). Since Sonazoid microbubbles accumulate in the liver parenchyma, the phase after 10 min or later is called the “post-vascular phase,” which is created by the accumulated microbubbles (12).

**Table 1:** Phases of contrast enhancement.

**Phase**	**Time from injection**	
	**Beginning**	**End**
Arterial phase	10–20 s	30–50 s
Portal phase	30–50 s	120 s
Late phase	>120 s	4–6 min
Postvascular phase^†^	10 min or later	

† The phase provided by using Sonazoid.

### Side effects

Although there are possible side effects with the use of microbubble contrast agents, the incidence is not high, is much lower than that with iodinated contrast materials, and may be comparable to that of contrast materials for magnetic resonance imaging (MRI) (10, 11). It is obviously important that CEUS should be performed in preparation for emergency/unexpected events.

## US based on circulating microbubbles

### Estimation of HVPG

The time difference of contrast onset between different vessels represents a parameter to estimate hepatic hemodynamics, initially reported by Albrecht et al. The parameter is called the transit time or arrival time, which depends on the severity of the liver disease (13).

Two studies have shown the effects of hepatic vein arrival time (HVAT) by CEUS with SonoVue; the area under the receiver operating characteristic curve (AUROC) was 0.973 under a cutoff value of 14 s for HVPG >10 mmHg with a sensitivity of 92.7%, a specificity of 86.7%, a positive predictive value of 90.5%, a negative predictive value of 89.7%, a positive likelihood ratio of 6.95, a negative likelihood ratio of 0.08 (14), and an AUROC of 0.72 under a cutoff value of 19 s for HVPG >12 mmHg with a sensitivity of 88.9% and a specificity of 58.1% to 62.8%. However, intrahepatic transit time provided much better diagnostic performance for HVPG >12 mmHg, AUROC of 0.94, sensitivity of 85.3–91.2%, and specificity of 77.8–88.9% with a cutoff value of 6 s, probably due to the exclusion of the influence of systemic circulation (15).

Splenic circulation was also effective in predicting HVPG, and CEUS with Sonazoid detected that the peak enhancement time (the interval time from the contrast onset in the splenic artery to the time to reach the maximum intensity level in the splenic vein) reflects the severity of PH, AUROC of 0.76 for HVPG ≥10 mmHg under a cutoff value of 13.5 s and an AUROC of 0.76 for HVPG ≥12 mmHg under the cutoff value of 14.5 s (16).

Subharmonic imaging (SHI) is a unique contrast-specific imaging technique that uses half of the transmission frequency with the advantage of improved tissue suppression. A significant correlation between subharmonic signal amplitude changes and portal venous pressure was observed in an animal study (17), and diagnostic abilities of 89% sensitivity and 88% specificity in patients with HVPG ≥10 mmHg and 100% sensitivity and 81% specificity in patients with HVPG ≥12 mmHg were reported in a clinical study (18). A more recent prospective clinical study demonstrated that the presence of optimized SHI signals inside the hepatic vein can be a qualitative screening test for PH with 83% reader agreement, which might reduce the need for invasive diagnostic procedures (19). In addition, the clinical significance of SHI for identifying clinically significant PH (CSPH) was supported in a systematic review of 45 studies of 5,678 patients; the AUROC of all imaging methods (acoustic radiation force impulse, CEUS, subharmonic-aided pressure estimation [SHAPE], liver stiffness by transient elastography [TE] or shear wave elastography, US, CT, and MRI) were >0.8, and TE, CEUS, and SHAPE exceeded 80% sensitivity and 80% specificity (20). Although the number of studies may not be sufficient, and the data are based on limited facilities, SHI may be a promising tool in the medical care of PH.

At this time, HVPG is only a surrogate marker of directly measured portal pressure, and the prediction of portal pressure may be meaningful. In this regard, a more recent study reported the correlation between portal pressure and parameters of CEUS, including hepatic artery-to-hepatic vein transit time (HA-HVTT), portal vein-to-hepatic vein transit time (PV-HVTT), and liver parenchyma-to-hepatic vein transit time (21). However, as the AUROC and cutoff value to detect specific pressure were not investigated in this study, the practical value of CEUS for assessing portal venous pressure needs to be further evaluated.

### Diagnosis of fibrosis/cirrhosis

A recent meta-analysis of 12 studies including 844 patients with chronic liver disease reported that the sensitivity, specificity, positive likelihood ratio, and negative likelihood ratio of HVAT measured by CEUS for the detection of cirrhosis compared to liver biopsy were 0.83, 0.75, 3.45, and 0.28, respectively. In addition, the summary diagnostic odds ratio (random effects model) was 15.23, and the summary AUROC was 0.74, suggesting increased diagnostic accuracy of the measurement of HVAT by CEUS for the detection of cirrhosis (22).

There is a study regarding another parameter of time interval; the interval time between the portal onset and the time of maximum intensity ratio between the right portal vein (RPV) and parenchyma with the use of Sonazoid provided a relationship with the hepatic fibrosis grade, showing AUROCs of 0.94 for ≥F2 with the cutoff value of 6.5 s, 0.96 for ≥F3 with the cutoff value of 8.0 s, and 0.98 for cirrhosis with the cutoff value of 9.5 s (23). The parameter means a time-related intensity change that has a relation to the microbubble distribution to the periphery. In fact, the maximum intensity ratio of the RPV to the liver parenchyma decreased according to the severity of the fibrosis, most likely because of the synergetic effect of the decreased filling rate in the RPV with microbubbles and rapid parenchymal enhancement because of arterialization. A subsequent study compared the diagnostic ability with the following four markers: contrast parameter [Sonazoid, the contrast parameter used in (23)], transient elastography (TE), type IV collagen 7s, and FIB-4 (age [years] × aspartate aminotransferase [U/L]/[platelet (10^9^/L) × alanine aminotransferase^1/2^ (U/L)]) (24). In a single model, the AUROCs were 0.83 for ≥F2 with FIB-4, 0.85 for ≥F3 with TE, and 0.92 with type IV collagen 7 s for cirrhosis. In contrast, in the combined model with a contrast parameter, the AUROCs were 0.87 for ≥F2 with FIB-4, 0.89 for ≥F3 with TE, and 0.99 for cirrhosis with TE.

According to the data using SHAPE to differentiate between mild (stage 0–1) and moderate to severe (stage 2–4) fibrosis in patients with dialysis, SHAPE succeeded (-10.4 ± 4.9 dB vs.−5.4 ± 3.2 dB; *P* = 0.035), but HVPG failed (3.0 ± 0.6 mmHg vs. 4.8 ± 0.7 mmHg; *P* = 0.30) (25). The diagnostic accuracy by SHAPE was 80%, whereas that by HVPG was 76%.

## US based on accumulated microbubbles

A US beam with a high MI induces an easy breakdown of microbubbles, and subtraction of the intensity before and after the breakdown may be useful for estimating the number of accumulated microbubbles (26). The post-vascular phase with the use of Sonazoid is suitable in this regard because the image originates from accumulated microbubbles with an absence or a minimum of circulating microbubbles. A prospective study of 203 subjects showed that intensity analysis with Sonazoid provided AUROCs 0.88 for ≥F2, 0.95 for ≥F3, and 0.97 for cirrhosis, which is useful for grading hepatic fibrosis (27). Furthermore, the performance was greater than that of FIB4 0.85 (≥F2, *P* = 0.15); 0.89 (≥F3, *P* = 0.057); 0.90 (cirrhosis, *P* = 0.017). However, the effect of accumulated microbubbles on the prediction of HVPG or portal venous pressure has not yet been determined.

## Management for the complications of PH

The detection and assessment of complications due to PH are essential for the practical care of patients. First, Li et al. focused on the detection of esophageal varices in patients with hepatitis B virus-related cirrhosis and found that the AUROC of intrahepatic transit time [difference between hepatic vein arrival time and hepatic artery arrival time (HV–HA)] for the assessment of the presence of esophageal varices and high-risk esophageal varices was 0.883 and 0.915, respectively, with a cutoff value of 8.2 s for the presence of esophageal varices and that of 7 s for the presence of high-risk esophageal varices (28). Portal vein thrombosis is also a common complication in patients with PH, with a risk of worsening portal hemodynamics (1–3). Investigators have shown the benefits of CEUS in the management of patients with portal vein thrombosis, the differential diagnosis between thrombus and tumor thrombus (29), and the prediction of anticoagulation outcomes (30, 31). CEUS is also applicable to the detection of portal hypertensive gastropathy by quantitative analysis of the contrast effect in the stomach wall with the use of Sonazoid (32) and to estimate the prognosis and occurrence of HCC in patients with cirrhosis by analyzing the time from hepatic arterial enhancement to maximum enhancement of the liver parenchyma, which was defined as the “hepatic filling rate” (33).

The CEUS is effective for the evaluation of shunt function in patients who undergo transjugular intrahepatic portosystemic shunt (TIPS), which is an essential therapeutic method for various complications of PH. Shunt dysfunction is a major event after the procedure and is the main issue that needs to be monitored. Although conventional color Doppler has been used to check the flow in the stent, recent studies have shown the effectiveness of CEUS in the assessment of shunt dysfunction by avoiding additional radiation exposure (34, 35). Although these data appear to support the benefit of CEUS over a broad area, they are only a part of cirrhosis-related complications, and there are limited data using CEUS. The substantial and comprehensive value of CEUS in this field needs to be validated in additional studies.

## Differential diagnosis between cirrhosis and idiopathic PH

Idiopathic PH (IPH) represents non-cirrhotic PH characterized by gastroesophageal varices, ascites, and portal vein thrombosis (36). Moreover, patients with IPH have clinical features of a lower incidence of HCC and a better prognosis than those with cirrhosis. Therefore, there is a significant difference in the clinical management of patients with IPH and those with cirrhosis. However, imaging tools such as CT and/or US are not necessarily effective in differentiating between them because of their similarities, atrophy and deformity of the liver, splenomegaly, and presence of ascites.

Three clinical studies have shown the effectiveness of CEUS with Sonazoid in differentiating between IPH and cirrhosis. First, a study using images from the arterial phase to the portal venous phase demonstrated the unique appearance of periportal delayed enhancement in the liver, which strongly suggests a diagnosis of IPH (37). The next study used post-vascular phase imaging and found that greater accumulation of intrahepatic microbubbles is a characteristic finding of IPH, analyzed by the intensity difference before and after high-power transmission (26). The last study reported the efficacy of 3D CEUS, which could demonstrate a unique and characteristic structure of the intrahepatic portal vein of IPH with a similar diagnostic ability to direct portography (38). Evidence strongly recommends the application of CEUS with Sonazoid when diagnosing IPH.

## Summary and limitations

The CEUS has great benefits in the practical medical care of patients with PH, prediction of HVPG, management of complications, and diagnosis of cirrhosis and non-cirrhotic PH, with the advantages of a simple procedure and less invasiveness, compared to liver biopsy and/or hepatic venous catheterization. Evidence has shown that HVAT is the parameter that should be primarily used to assess significant liver disease in daily medical care due to good reproducibility and diagnostic performance (9, 13–15, 22).

However, there are several limitations. First, the dependence on the skill and experience of the US operator may affect the demonstration and interpretation of the contrast-enhanced findings. Second, a definite methodology for imaging and suitable parameters have not been established for each purpose, probably due to the small number of high-quality studies. Third, the relative advantage of CEUS over other non-invasive imaging techniques for the detection of advanced liver fibrosis and CSPH has not been well-discussed. Thus, in everyday clinical practice, it should be recognized that the role of CEUS may be complementary to other non-invasive tools in this field, and it is recommended to be performed by trained operators to confirm the reproducibility of the data.

Unfortunately, the recent advancement of this field may be somewhat stagnant because of the lack of introduction of newer contrast agents, poor development of contrast-specific imaging mode and methodology of analysis.

Against this background, it is strongly recommended to organize future studies in (1) an international large-scale setting with cohorts from various backgrounds, (2) a comparison of diagnostic performance in the parameters between CEUS and other modalities followed by validation, and (3) active promotion of industry-academia joint research to enhance the development of new contrast agents with contrast-specific imaging methods.

## Conclusion

Although there are much positive data that suggest the benefit of CEUS in the management of PH, more studies with larger patient populations are required to confirm their substantial effect and cost-effectiveness. Continuous research may help develop an optimal CEUS parameter with high reliability and reproducibility.

## Author contributions

Conception and design: HM. Administrative support: TY. Provision of study materials or patients: MT. Collection and assembly of data: HN. Data analysis and interpretation: SS. All authors wrote the manuscript and approved the final manuscript.
